# Assessment of *FSHR*, *AMH*, and *AMHRII* variants in women with polycystic ovary syndrome

**DOI:** 10.1007/s12020-014-0345-4

**Published:** 2014-07-11

**Authors:** Ewa Czeczuga-Semeniuk, Katarzyna Jarząbek, Marzenna Galar, Piotr Kozłowski, Nela A. Sarosiek, Gabriela Zapolska, Sławomir Wołczyński

**Affiliations:** 1 Department of Reproduction and Gynecological Endocrinology, Medical University of Białystok, Skłodowskiej-Curie 24A, 15-276 Białystok, Poland; 2 Department of Hematology, Medical University of Białystok, Skłodowskiej-Curie 24A, 15-276 Białystok, Poland; 3 European Centre of Bioinformatics and Genomics (ECBaG), Institute of Bioorganic Chemistry, Polish Academy of Sciences, Noskowskiego 12/14, 61-704 Poznań, Poland; 4 Diagnostic Imaging and Radiology Department of J. Śniadecki Public Hospital Białystok, Skłodowskiej-Curie 26, 15-950 Białystok, Poland

Polycystic ovary syndrome (PCOS) is the most frequent endocrinopathy present in 6–15 % of reproductive-age women [1]. It is a heterogenous syndrome [2, 3] and according to the Rotterdam Criteria [4], PCOS is recognized in women who have two of three symptoms: chronic amenorrhea, clinical or biochemical hyperandrogenism and the ovarian morphology of PCO in ultrasound imaging, after other reasons have been excluded.

Disorders in the function of adipose tissue connected with adipocyte activity, that is, the secretion of the adipokines, adiponectin and retinoid binding protein 4 (RBP4), can be the main factors influencing the metabolic disorders frequently observed in PCOS women [5, 6].

The etiology of this disease has not been completely explained, thus far. Among factors impair the functioning of the hypothalamic-pituitary and ovary axis hormones and their receptors, the frequent genetic variant in form of single nucleotide polymorphisms (SNPs) was undoubtedly proven in genetic association studies to identify disease susceptibility loci. A genome-wide association study (GWAS) in Chinese women with PCOS confirmed that a region on chromosome 2p16.3 is associated with PCOS [7]. The results of a genetic association study published in 2013 confirmed that this region is also associated with PCOS in US Caucasian women [8]. Among the hundreds of SNPs which were localized in the *FSHR* gene, only five were identified in the coding region: in exon 10, at codons 307, 329, 524, 665, and 680. Of these, two (rs6165 and rs6166), which are in linkage disequilibrium [9], were characterized in various populations.

The anti-Müllerian hormone (AMH) produced in granulosa cells of follicles in ovary is also an important regulator of folliculogenesis, which may play a role in the pathophysiology of PCOS [10]. It has been proven that two genetic variants of the *AMH* gene and *AMHRII* receptor gene influence the internal sensitivity of the ovary to FSH and aromatase activity [11].

Therefore, in this context, we decided to investigate frequency of the SNPs in the *FSHR*, *AMH*, and *AMHRII* genes in a population of Polish women with PCOS, and to evaluate the possible association between these variants and susceptibility to PCOS and to concentrations of the adipokines, adiponectin, and RBP4.

## Materials and Methods

### PCOS and controls women

The study group included 294 premenopausal Caucasian patients with PCOS, and 78 women with regular menorrhea and without hirsutism. The body mass index (BMI) and the waist–hip ratio (WHR) was calculated. Hormonal examination (total testosterone—T, by immunoassay, Architect ci8200), hirsutism score ≥8 (according to the Ferriman–Gallwey scale, for cases mean value 9.80, SD 6.457) and ultrasound imaging were performed.

### DNA isolation and genotyping

Genomic DNA was isolated from 2 ml of peripheral EDTA-blood using the QIAamp Blood Midi Kit (Qiagen). Genotyping was performed using the TaqMan SNP Genotyping Assay on an Applied Biosystems 7500 System by Allelic Discrimination analysis. Four SNPs were studied: *FSHR* rs6165, rs6166, *AMH* rs10407022, and *AMHR II* rs2002555.

### Adiponectin and RBP4 assay

The concentrations of the retinol-binding protein 4 (RBP4) and the total adiponectin (total adiponectin/Acrp30) were determined in human serum by immunoenzymatic methods (ELISA) with the use of commercial Quantikine kits produced by R&D Systems (respectively: Quantikine ELISA Human RBP4 Immunoassay and Quantikine ELISA Human Total Adiponectin/Acrp30 Immunoassay). The optical density was determined using a microplate reader (Model 680 Microplate Readers, Bio-Rad Laboratories) and the Microplate Manager 5.2.1 software.

### Statistical analyses

Statistical analyses (for the clinical and biochemical characteristics) were performed using Mann–Whitney *U* test. Differences were considered to be significant at *P* < 0.05.

Association analyses were performed on the four genotyped SNPs. For all SNPs, the minor allele frequency (MAF) and genotype frequency (AA—frequent homozygote, AB—heterozygotes, BB—rare homozygotes) were calculated in the control and case groups. Prior to the association analysis, all SNPs were tested for agreement with the Hardy–Weinberg equilibrium (HWE) in the both groups, with the use of a χ^2^ test. All SNPs passed the HWE test. For these SNPs, we calculated the odds ratio (OR) with a 95 % confidence interval (CI), assuming the dominant model of inheritance; and the significance of the association was calculated using the χ^2^ test. For the SNPs with MAF >0.3, we also tested the codominant model of association with the use of the χ^2^ test for the trend.

For analysis of SNPs association with PCOS, we set the significance level of the α at 0.05. The levels of the adiponectin and RBP4 were compared in the control versus case groups, with the use of the two-tailed *t* test. The levels of adiponectin and RBP4 were also compared for all tested SNPs in the AA group versus the combined AB + BB groups, separately, in the case and control groups. The adiponectin and RBP4 levels were compared using the Pearson correlation analysis. All statistical analyses were performed using STATISTICA (StatSoft; Tulsa, OK, USA) or Prism v. 4.0 GraphPad Software (San Diego, CA, USA).

## Results

The characteristics of the patients included in the study are presented in Table 1.

**Table 1:** Characteristics of PCOS women and controls subjects

	Age (years)	Weight (kg)	BMI (kg/m^2^)	WHR	RBP4 (ng/ml)	Adiponectin (ng/ml)
Cases						
*N*	294	294	294	292	286	285
Mean value	24.84	68.68	24.73	0.81	31.39	119.24
SD	4.36	16.00	5.71	0.08	8.59	70.29
Median	24.00	65.00	23.10	0.80	30.14	104.75
Minimum	17.00	39.00	14.50	0.53	15.18	8.82
Maximum	42.00	138.00	53.90	1.06	86.11	361.68
Controls						
*N*	78	78	78	78	78	78
Mean value	23.17	61.40	21.61	0.75	27.79	147.22
SD	1.55	10.57	3.11	0.08	11.05	57.55
Median	23.50	60.00	21.00	0.75	26.72	138.11
Minimum	19.00	45.00	15.90	0.66	11.17	39.14
Maximum	27.00	95.00	31.60	1.24	85.77	329.74
*P*	*P* = 0.003	*P* < 0.001	*P* < 0.001	*P* < 0.001	*P* < 0.001	*P* < 0.001

The results obtained for the genotype distribution and allele frequencies proved that only the SNP rs10407022 in the gene *AMH*, is significantly associated with the decrease of the risk of the disease (*P* = 0.041, OR 0.58, 95 % CI 0.44–0.97). For two SNPs, rs10407022 and rs2002555, there were statistically significant differences in the genotype frequencies between patients with different combination of main three symptoms of PCOS (*P* < 0.009 and *P* < 0.037, respectively).

The marginally significant association (*P* = 0.014) with the adiponectin concentration (mean value of 167.9 in the AA group versus 135.0 in the BB + AB group) was shown for rs6165. There were no correlations between the adiponectin and RBP4 levels in the PCOS and in the control groups.

## Discussion

The clinical and biochemical parameters analyzed differed statistically significantly between the women with PCOS and the controls. Similar to the results obtained by Carmina et al. [12] in PCOS group we also observed higher mean values of RBP4 and the lowest mean values of adiponectin.

From all analyzed SNPs, rs6165, and rs6166 had the highest MAF in Polish population. These two SNPs, located in exon 10 of the *FSHR* gene were associated with PCOS susceptibility in the Japanese population [13]. Also, rs6165 was nominally associated with PCOS in women of European ancestry [8]. Furthermore, there was significant association between rs6166, haplotype G/A in *FSHR* gene and PCOS in the Han Chinese women [14]. However, the sample size of that study was relatively small. On the other hand, a study conducted on a larger population of Northern Chinese Han women demonstrated that Ser680 variants might be related to high FSH levels in that population, and both genetic variants, rs6165, rs6166 were not a causative factor of PCOS [15]. In this study, we did not find any differences in genotype and allele frequencies of these two variants between the case and the control groups. In addition, it was not observed any association between rs6165, rs6166, and PCOS in Polish women. There was only a slight association of rs6165 with the adiponectin values in the control group, but we did not prove this for the PCOS group.

In our control group, we did not confirm the genotype frequency of rs10407022 (*AMH* gene) obtained by Kevenaar et al. 2007 in the Dutch and German populations. Nevertheless, the *AMHRII* genotype frequency distribution in our normo-ovulatory and PCOs groups was in agreement with this and the latter report of Kevenaar et al. [16]. Examining the probable role of these genetic variants in the pathophysiology of PCOS, the same researchers proved that the SNPs examined had no influence on the risk of PCOS. Rs10407022 (*AMH* gene) did not contribute to the risk of PCOS, and the polymorphic *AMHRII* gene had no effect on the risk or the final phenotype of the syndrome. However, polymorphisms of the *AMH* gene influenced the severity of the phenotypic picture.

Our present research proved that the rarer SNP alleles of the *AMH* rs10407022 gene decreased the risk of the disease so we indicated that this polymorphic variant contributes in the pathogenesis of PCOS, but these findings must be replicated in larger cohorts of Polish women.

## References

[1] Fauser BC, Tarlatzis BC, Rebar RW, Legro RS, Balen AH, Lobo R, Carmina E, Chang J, Yildiz BO, Laven JS, Boivin J, Petraglia F, Wijeyeratne CN, Norman RJ, Dunaif A, Franks S, Wild RA, Dumesic D, Barnhart K (2012). Consensus on women’s health aspects of polycystic ovary syndrome (PCOS): the Amsterdam ESHRE/ASRM-Sponsored 3rd PCOS Consensus Workshop Group. Fertil. Steril..

[2] Witchel SF, Recabarren SE, González F, Diamanti-Kandarakis E, Cheang KI, Duleba AJ, Legro RS, Homburg R, Pasquali R, Lobo RA, Zouboulis CC, Kelestimur F, Fruzzetti F, Futterweit W, Norman RJ, Abbott DH (2012). Emerging concepts about prenatal genesis, aberrant metabolism and treatment paradigms in polycystic ovary syndrome. Endocrine.

[3] Deniz R, Gurates B, Aydin S, Celik H, Sahin I, Baykus Y, Catak Z, Aksoy A, Citil C, Gungor S (2012). Nesfatin-1 and other hormone alterations in polycystic ovary syndrome. Endocrine.

[4] Revised 2003 consensus on diagnostic criteria and long-term health risks related to polycystic ovary syndrome (PCOS). Rotterdam ESHRE/ASRM-sponsored PCOS consensus workshop group. Hum. Reprod. **19**, 41–47 (2004) 10.1093/humrep/deh098 14688154

[5] Yildiz BO, Bozdag G, Otegen U, Harmanci A, Boynukalin K, Vural Z, Kirazli S, Yarali H (2010). Visfatin and retinol-binding protein 4 concentrations in lean, glucose-tolerant women with PCOS. Reprod. Biomed. Online.

[6] Toulis KA, Goulis DG, Farmakiotis D, Georgopoulos NA, Katsikis I, Tarlatzis BC, Papadimas I, Panidis D (2009). Adiponectin levels in women with polycystic ovary syndrome: a systematic review and a meta-analysis. Hum. Reprod. Update.

[7] Chen ZJ, Zhao H, He L, Shi Y, Qin Y, Shi Y, Li Z, You L, Zhao J, Liu J, Liang X, Zhao X, Zhao J, Sun Y, Zhang B, Jiang H, Zhao D, Bian Y, Gao X, Geng L, Li Y, Zhu D, Sun X, Xu JE, Hao C, Ren CE, Zhang Y, Chen S, Zhang W, Yang A, Yan J, Li Y, Ma J, Zhao Y (2011). Genome-wide association study identifies susceptibility loci for polycystic ovary syndrome on chromosome 2p16.3, 2p21 and 9q33.3. Nat. Genet..

[8] Mutharasan P, Galdones E, Peñalver Bernabé B, Garcia OA, Jafari N, Shea LD, Woodruff TK, Legro RS, Dunaif A, Urbanek M (2013). Evidence for chromosome 2p16.3 polycystic ovary syndrome susceptibility locus in affected women of European ancestry. J. Clin. Endocrinol. Metab..

[9] Simoni M, Gromoll J, Nieschlag E (1997). The follicle-stimulating hormone receptor: biochemistry, molecular biology, physiology, and pathophysiology. Endocr. Rev..

[10] Durlinger AL, Visser JA, Themmen AP (2002). Regulation of ovarian function: the role of anti-Müllerian hormone. Reproduction.

[11] Kevenaar ME, Themmen AP, Laven JS, Sonntag B, Fong SL, Uitterlinden AG, de Jong FH, Pols HA, Simoni M, Visser JA (2007). Anti-Müllerian hormone and anti-Müllerian hormone type II receptor polymorphisms are associated with follicular phase estradiol levels in normo-ovulatory women. Hum. Reprod..

[12] Carmina E, Bucchieri S, Mansueto P, Rini G, Ferin M, Lobo RA (2009). Circulating levels of adipose products and differences in fat distribution in the ovulatory and anovulatory phenotypes of polycystic ovary syndrome. Fertil. Steril..

[13] Sudo S, Kudo M, Wada S, Sato O, Hsueh AJ, Fujimoto S (2002). Genetic and functional analyses of polymorphisms in the human FSH receptor gene. Mol. Hum. Reprod..

[14] Du J, Zhang W, Guo L, Zhang Z, Shi H, Wang J, Zhang H, Gao L, Feng G, He L (2010). Two FSHR variants, haplotypes and meta-analysis in Chinese women with premature ovarian failure and polycystic ovary syndrome. Mol. Genet. Metab..

[15] Fu L, Zhang Z, Zhang A, Xu J, Huang X, Zheng Q, Cao Y, Wang L, Du J (2013). Association study between FSHR Ala307Thr and Ser680Asn variants and polycystic ovary syndrome (PCOS) in Northern Chinese Han women. J. Assist. Reprod. Genet..

[16] Kevenaar ME, Laven JS, Fong SL, Uitterlinden AG, de Jong FH, Themmen AP, Visser JA (2008). A functional anti-mullerian hormone gene polymorphism is associated with follicle number and androgen levels in polycystic ovary syndrome patients. J. Clin. Endocrinol. Metab..

